# Pan-Bcl-2 inhibitor Obatoclax is a potent late stage autophagy inhibitor in colorectal cancer cells independent of canonical autophagy signaling

**DOI:** 10.1186/s12885-015-1929-y

**Published:** 2015-11-19

**Authors:** Bruno Christian Koehler, Adam Jassowicz, Anna-Lena Scherr, Stephan Lorenz, Praveen Radhakrishnan, Nicole Kautz, Christin Elssner, Johanna Weiss, Dirk Jaeger, Martin Schneider, Henning Schulze-Bergkamen

**Affiliations:** National Center for Tumor Diseases, Department of Medical Oncology, Internal Medicine VI, Heidelberg University Hospital, Heidelberg, Germany; Department of General, Visceral and Transplantation Surgery, University of Heidelberg, Im Neuenheimer Feld 110, 69120 Heidelberg, Germany; Department of Clinical Pharmacology and Pharmacoepidemiology, University Hospital Heidelberg, University of Heidelberg, Im Neuenheimer Feld 410, 69120 Heidelberg, Germany; Department of Internal Medicine II, Marien-Hospital, Wesel, Germany

**Keywords:** Autophagy, Colorectal cancer, Apoptosis, Autophagy related gene, LC3, p62 (SQSTM1), Obatoclax, Chloroquine

## Abstract

**Background:**

Colorectal cancer is the third most common malignancy in humans and novel therapeutic approaches are urgently needed. Autophagy is an evolutionarily highly conserved cellular process by which cells collect unnecessary organelles or misfolded proteins and subsequently degrade them in vesicular structures in order to refuel cells with energy. Dysregulation of the complex autophagy signaling network has been shown to contribute to the onset and progression of cancer in various models. The Bcl-2 family of proteins comprises central regulators of apoptosis signaling and has been linked to processes involved in autophagy. The antiapoptotic members of the Bcl-2 family of proteins have been identified as promising anticancer drug targets and small molecules inhibiting those proteins are in clinical trials.

**Methods:**

Flow cytometry and colorimetric assays were used to assess cell growth and cell death. Long term 3D cell culture was used to assess autophagy in a tissue mimicking environment in vitro. RNA interference was applied to modulate autophagy signaling. Immunoblotting and q-RT PCR were used to investigate autophagy signaling. Immunohistochemistry and fluorescence microscopy were used to detect autophagosome formation and autophagy flux.

**Results:**

This study demonstrates that autophagy inhibition by obatoclax induces cell death in colorectal cancer (CRC) cells in an autophagy prone environment. Here, we demonstrate that pan-Bcl-2 inhibition by obatoclax causes a striking, late stage inhibition of autophagy in CRC cells. In contrast, ABT-737, a Mcl-1 sparing Bcl-2 inhibitor, failed to interfere with autophagy signaling. Accumulation of p62 as well as Light Chain 3 (LC3) was observed in cells treated with obatoclax. Autophagy inhibition caused by obatoclax is further augmented in stressful conditions such as starvation. Furthermore, our data demonstrate that inhibition of autophagy caused by obatoclax is independent of the essential pro-autophagy proteins Beclin-1, Atg7 and Atg12.

**Conclusions:**

The objective of this study was to dissect the contribution of Bcl-2 proteins to autophagy in CRC cells and to explore the potential of Bcl-2 inhibitors for autophagy modulation. Collectively, our data argue for a Beclin-1 independent autophagy inhibition by obatoclax. Based on this study, we recommend the concept of autophagy inhibition as therapeutic strategy for CRC.

**Electronic supplementary material:**

The online version of this article (doi:10.1186/s12885-015-1929-y) contains supplementary material, which is available to authorized users.

## Background

Colorectal tumors are one of the major causes for cancer related death in humans [1]. Even if novel and targeted therapeutic approaches are rapidly emerging, the prognosis in the metastatic stage (UICC IV) is restricted.

Autophagy is an evolutionarily conserved process of cellular self-digestion, which is indispensable in situations of cellular stress such as hypoxia, energy deprivation or an acidic environment [2]. In gut development and pathophysiology, autophagy plays a decisive role [3]. An imbalance within the tightly regulated autophagy network has implications for various human diseases including cancer [4, 5]. It has been proven in various cancer models that autophagy represents an important mechanism by which cancer cells maintain their highly active metabolism [6, 7]. Furthermore, autophagy may provide resistance towards antitumor therapy [8]. The exact mechanism by which autophagy modulates malignant transformation has been a matter of controversy in current research [7, 9].

By now, there is a huge and growing body of preclinical and clinical data investigating genetic or chemical approaches to block autophagy as a therapeutic strategy in cancer [6, 10, 11]. Potent autophagy inhibitors such as the malaria drug chloroquine have entered late stage clinical trials [12]. Little is known regarding autophagy inhibition in colorectal cancer. Studies present heterogeneous data regarding autophagy in CRC [13–15].

The Bcl-2 family of proteins is mainly known for its pivotal role in the regulation of mitochondrial apoptosis [16]. A variety of small molecules targeting antiapoptotic Bcl-2 proteins have been developed with the aim to overcome cell death resistance in cancer [17]. Several Bcl-2 inhibitors have entered clinical trials [17, 18]. In addition, it has been shown that antiapoptotic Bcl-2 proteins interfere with autophagy signaling. For instance, Bcl-2 binds, among others, to proautophagic Beclin-1, thereby blocking autophagy induction [19, 20].

In order to prove the feasibility and potency of autophagy inhibition as concept for colorectal cancer treatment, this study first aimed at investigating chloroquine in CRC cells as a model agent for autophagy inhibition.

There are several Bcl-2 mimicking small molecules available. In this study we investigated pan Bcl-2 inhibition by obatoclax compared to the Mcl-1 sparing inhibitor ABT-737. We have recently shown that the pan-Bcl-2 inhibitor obatoclax induces cell cycle arrest and blocks migration in CRC cells rather than causing direct cell death, arguing for pleiotropic antitumor effects of this agent [21]. Since others have reported a contribution of obatoclax to autophagy signaling in cancer, we focused on autophagy in CRC cells treated with obatoclax [22, 23]. Therefore, the aim of this study was to further dissect the anti-cancer properties of obatoclax in colorectal cancer cells and to further elucidate the contribution of antiapoptotic Bcl-2 proteins in the crosstalk between apoptosis and autophagy.

## Methods

### Reagents and cell lines

The colorectal cancer cells HT29 and SW480 were obtained from ATCC and cultured under standard conditions as described previously, supplemented with 10 % fetal bovine serum (PAA laboratories, Cölbe, Germany) in a humidified atmosphere [21]. Starvation was induced using OptiMEM (Invitrogen, Karlsruhe, Germany) as a reduced medium without supplements.

Chloroquine was purchased from Sigma-Aldrich (Hamburg, Germany). Obatoclax and ABT-737 were obtained from Selleckchem (Munich, Germany), staurosporine was from Enzo Life Science (Farmingdale, NY, USA).

### RNA-interference and transfection

Transfections were carried out in OptiMEM without supplements using Lipofectamine RNAi-Max (Invitrogen, Karlsruhe, Germany) as described [24]. Sequences for siRNA targeting Mcl-1, Beclin-1, Atg7 and Atg12 are available upon request; a non-targeting siRNA (siScramble) was used as a control (MWG Biotech, Ebersberg, Germany). 24 h post transfection cells were treated for 48 h

### Cell death and growth assays

Flow cytometry analyses were performed using a FACS CANTO II (Becton Dickinson, Franklin Lakes, NJ, USA) as described previously [21]. Cells in the sub-G1 fraction were depicted as apoptotic.

Cell growth was assessed using a colorimetric 3-(4, 5-dimethylthiazol-2-yl)-2, 5-diphenyltetrazolium bromide (MTT) based assay as described previously [25]. Absorbance was measured at 550 nm using a plate reader (Infinite 200 pro; Tecan, Männedorf, Switzerland). Values were normalized to untreated controls.

### Immunohistochemistry and 3D cell culture

Sections (8 μm thickness) were fixed in 4 % PFA and stained with haematoxylin and eosin according to standard procedures. Immunohistochemistry was performed using NovoLink Polymer detection System (Leica Microsystems, Wetzlar, Germany) according to the manufacturer’s instructions. Images were captured using an inverted microscope (Keyence, Neu-Isenburg, Germany). Images were analyzed using CellSense® and ImageJ software.

Long term cell culture in 3-dimensional ALVETEX scaffolds (Reinnervate, Sedgefield, UK) fosters cell-cell-interactions in a tissue mimicking environment and has been described previously [24]. Fresh medium was added every 48 h. After 7 days of treatment, scaffolds were collected, shock frozen and subsequently sectioned and stained as described [24]. The following antibodies were used for detection: anti-LC3b (Cell Signaling, Boston, MA, USA) anti-p62 (BD, Franklin Lakes, NJ, USA).

### SDS-Page, western blotting and q-RT PCR

Cell lysis, SDS-page and western blotting were performed according to standard procedures as described previously [21]. The following antibodies were used: anti-Mcl-1 (Santa Cruz Biotechnology, Heidelberg, Germany), anti-LC3b (Cell Signaling), anti-p62 (BD), anti-Atg12 (Cell Signaling), anti-Atg7 (Cell Signaling), anti-Tubulin (Sigma, St. Louis, MO, USA.

RNA isolation, cDNA synthesis and quantitative real time polymerase chain reaction (q-RT PCR) were carried out as described using primer assay kits [21]. Each reaction was run in duplicates, values were normalized to GAPDH as housekeeping gene.

### Supravital cell-staining with acridine orange

Cells grown on cover slips were treated as described. 24 h following initial treatment, cells were incubated with medium containing 2 μg/mL acridine orange (Sigma-Aldrich) for 20 min. Subsequently, medium containing acridine orange was removed and cells were washed twice with PBS and fixed using 4 % PFA (Merck) for 15 min at room temperature. Pictures were obtained using a Zeiss LSM 780 confocal laser scanning microscope with ZEN microscopy software at 63x magnification.

### Immunofluorescence

Cells grown on cover slips were treated as described above. 24 h following initial treatment, cells were washed twice with PBS and fixed using ice-cold methanol at −20 °C for 20 min. Fixed cells were washed twice with PBS and blocked in PBS containing 5 % BSA and 0.3 % Triton™ X-100 (Merck) for 1 h. After blocking, cells were incubated with primary antibodies in PBS with 1 % BSA and 0.3 % Triton™ X-100 at 4 °C overnight. The next day, cells were incubated with Alexa Fluor 488-conjugated goat anti-rabbit secondary antibodies (Invitrogen, Carlsbad, California, USA) for 1 h at room temperature and subsequently counterstained with PBS containing 2 μg/mL Hoechst 33342 trihydrochloride trihydrate (Invitrogen) for 15 min at room temperature. Pictures were obtained using a Zeiss LSM 780 confocal laser scanning microscope with ZEN microscopy software at 63 × magnification and 10 x magnification for counting of LC3II puncta. The average of 5 visual fields at 10 x magnification was used.

### In vitro lysosomal staining

Cells grown in 12-well cell-culture plates were treated as described above. 24 h following initial treatment, cells were washed twice with PBS and incubated with reaction buffer containing 2 μL Cyto-ID® Green Detection Reagent (Enzo), 1 μL Hoechst 33342, Trihydrochloride, Trihydrate (Enzo) and 5 % FCS per mL reaction buffer for 25 min at 37 °C in a cell incubator [26]. Subsequently, the reaction buffer was removed, cells were washed and fresh reaction buffer was added. Cell-culture plates were analyzed using a Biorevo BZ-9000 Inverted fluorescence phase-contrast microscope with BZ-observer microscopy software (Keyence) at 40x magnification.

### Statistical analysis

Statistical analysis was done using student’s *t-test* (paired, two sided) based on normal data distribution. All statistics were calculated using SPSS 20 (IBM, NY, USA), p-values < 0.05 were considered to be significant.

## Results

### Late stage autophagy inhibition induces apoptosis in starved CRC cells

Chloroquine (CQ) has been widely used as a model agent for autophagy inhibition [27, 28]. Here, we demonstrate that CQ has limited impact on HT29 and SW480 CRC cells in fully supplemented growth medium. In contrast, CQ is able to induce cell death in CRC cells under conditions of starvation (Additional file 1: Figure S1 A left and right graph, *p* < 0.001).

In order to investigate autophagy signaling upon CQ treatment, we assessed protein levels of Light Chain Enhancer 3 (LC3) and the conversion from soluble LC3I to membrane-bound LC3II, which is indicative for autophagy flux activation (Additional file 1: Figure S1 B) [29, 30]. In addition, we observed accumulation of p62, also termed sequestosome 1 (SQSTM1) in cells under CQ treatment. P62 gets degraded in autophagic vesicles and is therefore an indicator of autophagic flux [31, 32]. Lysotracker and acridine orange (AO) staining revealed marked cytosolic accumulation of acidic vesicles in HT29 and SW480 cells (Additional file 1: Figure S1 C and Additional file 2: Figure S2) [33–35]. Of note, hypoxia and cell death induced by staurosporine (STS) failed to modulate autophagy as shown in Additional file 1: Figure S1 B.

### Obatoclax inhibits cell growth and induces cell death in autophagy promoting conditions

**Fig. 1:**
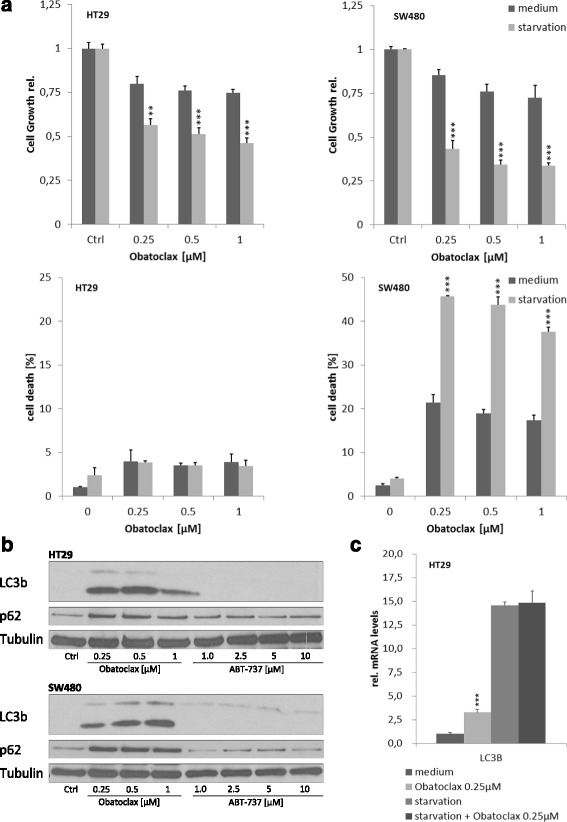
Obatoclax blocks cell growth and induces apoptosis in starving CRC cells. **a** and **b** HT29 and SW480 cells were treated with obatoclax in full supplemented medium or starvation as indicated. **a** Cell growth was assessed by MTT-Assay after 48 h. Cell death was assessed by Flow Cytometry after 48 h. Assays were done in triplicates. Values are expressed as mean ± SD. **b** Representative Western blots for p62 and LC3 I/II in HT29 and SW480 treated with obatoclax or ABT-737 in full supplemented medium as indicated. **c** Rel. mRNA levels of LC3 in HT29 cells after 24 h treatment with obatoclax. mRNA levels were quantified by qRT-PCR and normalized to GAPDH as housekeeping gene. Assays are representative of at least three independent experiments . ** = *p* < 0.01; *** = *p* < 0.001

In starving cells, catabolic autophagy becomes crucial to keep cell metabolism running. Our data show that obatoclax-induced growth inhibition in SW480 and HT29 colorectal cancer cells is profoundly augmented under starvation (Fig. 1a, left and right upper graphs). Of note, starvation alone did not reduce growth of HT29 and SW480 cells (data not shown).

HT29 cells are resistant to obatoclax-induced cell death even under starving conditions. By contrast, SW480 cells show a high sensitivity towards obatoclax, which is strikingly enhanced upon starvation. The amount of propidium iodide (PI) positive SW480 cells increased from 21 % to 46 % when exposed to starvation instead of fully supplemented medium after 48 h obatoclax treatment (Fig. 1a, *p* < 0.001).

In order to investigate the role of obatoclax in autophagy of CRC cells, we performed western blot analyses. We show that obatoclax, but not ABT-737, induces profound inhibition of autophagy in CRC cells pointing to a unique feature of obatoclax regarding autophagy regulation. As shown in Fig. 1b, immunoblotting reveals a massive obatoclax induced accumulation of LC3 in both cell lines, as well as an increased conversion from LC3I to LC3II. This effect is already induced by sublethal doses. In addition, we observed p62 accumulation in cells treated with obatoclax. Collectively, the coinciding accumulation of LC3II and p62 indicates a late stage autophagy inhibition by obatoclax.

Next, we sought to explore whether the impact of obatoclax on autophagy relevant proteins is limited to the protein level. Q-RT PCR revealed an increase of LC3 mRNA transcription in obatoclax treated cells (Fig. 1c). This effect holds true under starvation.

### Obatoclax is a late stage autophagy inhibitor in CRC cells

**Fig. 2:**
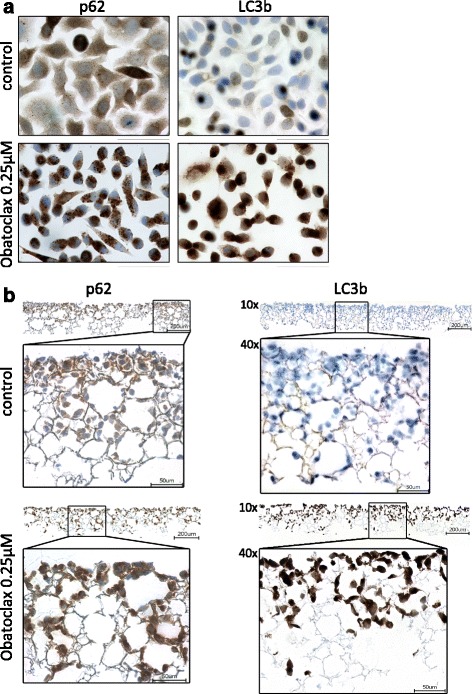
Autophagy regulation by Obatoclax in 3D long term cell culture. **a** HT29 cells were seeded on glass slides, grown for 7 days and treated with 0.25 μM obatoclax. Immunhistochemical staining was done for p62 and LC3b. **b** HT29 cells in scaffolds after 7 days treatment with obatoclax. Immunhistochemical staining for p62 and LC3b. Representative pictures for at least 3 experiments are shown. Scale bar indicates 200 μm for longitudinal sections and 50 μM for corresponding insets. DMSO served as vehicle

In order to validate our observations made by immunoblotting and to investigate the subcellular distribution of autophagy relevant proteins, we performed immunohistochemistry of cells treated with obatoclax. Two different cell growth conditions were applied to study LC3 and p62 in obatoclax treated CRC cells. First, we seeded and cultured HT29 on glass slides and applied obatoclax treatment for 24 h. In mock treated control cells, there was only a marginal amount of LC3 detectable. In line with our findings from western blots, we observed a striking accumulation of LC3 in cells treated with obatoclax (Fig. 2a, right). P62 staining revealed ubiquitous expression in control cells. The staining was cytosolic and homogeneous in a granular fashion. In comparison, obatoclax treatment caused a much more intense staining with emphasized large granules (Fig. 2a, left).

3D polystyrene scaffolds foster cell-cell-interaction mimicking tissue growth. Furthermore long-time studies are feasible within this in vitro system. In line with our earlier reports, obatoclax reduced cell growth and decelerated migration (data not shown). LC3 was not detectable in untreated control cells but showed massive accumulation after long term obatoclax treatment. In sections of polystyrene scaffolds p62 showed a weak expression in mock treated cells. By contrast, strong cytosolic signal for p62 was detected in cells after 7 days of obatoclax treatment (Fig. 2b). In summary, these observations point towards a swift but sustained late stage autophagy blockade.

In order to visualize the autophagic flux in cells treated with obatoclax, we applied acridine orange (AO) staining. In untreated cells the unprotonated form of AO shows a homogeneous cytosolic distribution (Additional file 2: Figure S2). Under obatoclax treatment, the protonated form becomes visible in a granular stain with a predominantly perinuclear localization. This observation augments our hypothesis that autophagosomes are built and formed but not degraded anymore in cells treated with obatoclax (Additional file 2: Figure S2).

**Fig. 3:**
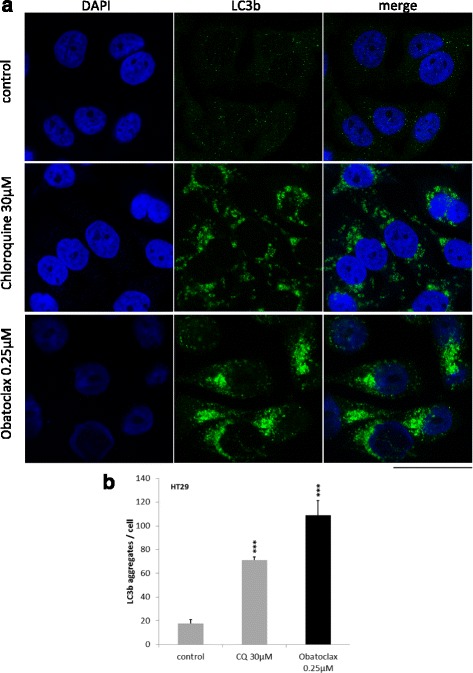
LC3 accumulates in autophagosomes in cells treated with Obatoclax. **a** Immunofluorescence staining for LC3 in untreated (upper panel), Chloroquine treated (30 μM, middle panel) and obatoclax treated (0.25 μM, lower panel) cells. Representative pictures are shown. Assays were done at least three times. **b** Analysis of LC3 aggregates per cell. Values are expressed as mean ± SD. *** = *p* < 0.001

CQ has been introduced as a model agent for autophagy inhibition (Additional file 1: Figure S1). We stained obatoclax and CQ treated HT29 cells with a fluorophore labeled antibody against LC3. Mock treated cells showed virtually no positivity for LC3. In comparison to CQ, obatoclax caused a much more intense fluorescence signal by means of cytosolic LC3 puncta (Fig. 3a and b, *p* < 0.001).

### Obatoclax blocks autophagy independently of Beclin-1, Atg7 and Atg12

**Fig. 4:**
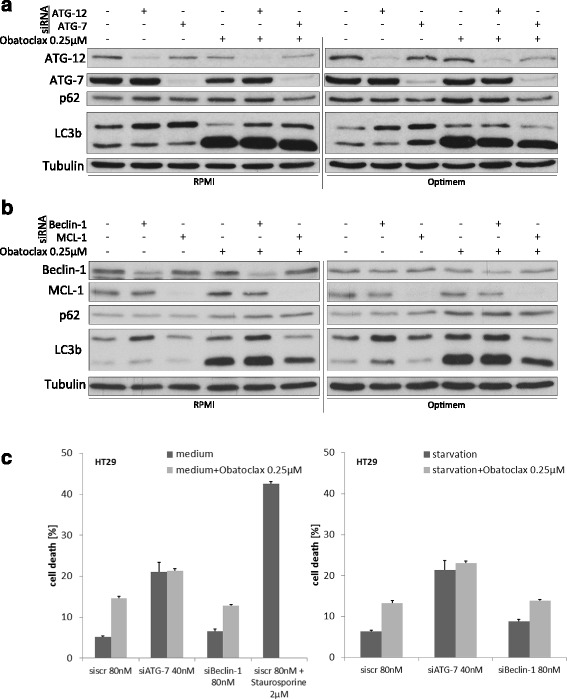
Obatoclax blocks autophagy independent of canonical autophagy signaling. **a** and **b** Representative western blotting for HT29 cells treated with obatoclax. siRNA mediated knockdown of Atg12 and Atg7 is shown in (**a**), siRNA mediated knockdown of Mcl-1 or Beclin-1 is shown in (**b**). **c** Flow cytometric analysis for apoptosis induction in full supplemented (left) or starving (right) HT29 cells after knockdown of Atg7 or Beclin-1 and treatment with obatoclax. Values are expressed as mean ± SD. Staurosporine 2 μM for 2 h served as a positive control for cell death induction

Autophagy related gene (Atg) 7 and Atg12 mediate important early steps during the formation of the autophagosome downstream of Beclin-1. In untreated cells, siRNA mediated downregulation of Atg7 or Atg12 prevents LC3 conversion, leading to an accumulation of the soluble LC3I form. Nevertheless, the obatoclax-induced increase of LC3II was unaltered after Atg7 or Atg12 knockdown. Of note, p62 expression is weaker in obatoclax treated cells after Atg7 knockdown (Fig. 4a). The independence of obatoclax effects from Atg7 and Atg12 held true for cells under fully supplemented and starving conditions.

Beclin-1 is a central pro-autophagic protein involved in early autophagosome biogenesis. Moreover, it has been demonstrated that Beclin-1 interacts with antiapoptotic Bcl-2 and Bcl-x_L,_ representing a key switch between apoptosis and autophagy signaling. We therefore decided to investigate the role of Beclin-1 for the autophagy inhibition caused by obatoclax. siRNA mediated knockdown of Beclin-1 led to high expression of LC3I. The conversion from LC3I to LC3II was insufficient after knockdown of Beclin-1 (Fig. 4b). Furthermore, we did not observe an accumulation of p62. The described pattern of autophagy relevant proteins is indicative for an early block of autophagy caused by downregulation of Beclin-1 (Fig. 4b). Impressively, obatoclax treatment led to a massive accumulation of both, p62 and LC3II, even after knockdown of Beclin-1.

We showed a starvation-dependent cell death induction caused by obatoclax in SW480 cells (Fig. 1). However, we further investigated cell death under obatoclax treatment after knockdown of Atg7 and Beclin-1. We observed an unchanged cell death induction pattern in cells treated with obatoclax after RNAi mediated knockdown of either Atg7 or Beclin-1 (Fig. 4 c).

### Mcl-1 is a regulator of autophagy in colorectal cancer cells

Since we could show that ABT-737 failed to interfere with autophagy (Fig. 1), we further focused on Mcl-1 and investigated its role in autophagy and its importance for the effects of obatoclax. A siRNA mediated knockdown of Mcl-1 did not influence autophagy signaling as shown by an unchanged expression of LC3I and LC3II as well as p62 in untreated cells (Fig. 4b). Intriguingly, knockdown of Mcl-1 led to an impaired autophagy inhibition in HT29 cells, treated with obatoclax. The impaired effectiveness of obatoclax on autophagy becomes obvious in a decreased expression of LC3I and a reduced conversion from LC3I to LC3II (Fig. 4b).

## Discussion

Autophagy has a decent role in colorectal organogenesis, mucosal homeostasis and disease development. In colorectal cancer onset, autophagy plays a protective role for cancer cells by means of energy delivery. However, targeting autophagy has been recently discovered as promising and novel treatment approach [9, 36]. Autophagy is regulated by a tightly balanced signaling network of enormous complexity. Within the autophagy signaling network, crosstalks with classical apoptosis pathways have been identified [37, 38]. For instance, Bcl-2, an important antiapoptotic protein, binds proautophagic Beclin-1 thereby inhibiting autophagy [20, 39].

First, we explored the potential impact of autophagy inhibition in CRC cells. The malaria drug chloroquine inhibits the execution of autophagy at a late stage via an elusive mechanism [40]. Our data indicates that CQ is an effective autophagy inhibitor in CRC cells. A moderate apoptosis induction by CQ is massively augmented in cells under starvation. Hypoxia and direct autophagy induction via Staurosporine failed to regulate autophagy. This observation argues for a pivotal role of autophagy and substantiates our hypothesis that inhibition of autophagy can trigger apoptosis induction in CRC.

In our present study, we sought to investigate the role of Bcl-2 inhibition for autophagy. There are various small molecules available, mimicking proapoptotic proteins, which may lead to apoptosis induction. We have recently shown that the pan-Bcl-2 inhibitor obatoclax blocks invasiveness of colorectal cancer cells and causes cell cycle arrest in G1-Phase [21]. Furthermore our earlier studies demonstrated that Mcl-1 sparing ABT737 but not obatoclax induced apoptotic cell death in CRC cells. Here, we show that cell growth inhibition as well as cell death induction caused by obatoclax is profoundly augmented when cells are challenged by starvation. The degree of cell death induction and the subtype of death in this context depend on the cell type. HT29 compared to SW480 cells are likely to die through necroptosis rather than apoptosis [41].

This observation suggests a major role of autophagy, since it becomes crucial for cell survival and growth in stressful situations. Immunoblotting showed a massive upregulation of LC3 on the protein level and an increased shift from LC3I to the activated and membrane bound form LC3II. The increase of LC3 was accompanied by an accumulation of p62, a scaffold protein of autophagic vesicles, in cells treated with obatoclax. This protein pattern is indicative for a late stage inhibition of autophagy, because high levels of LC3 indicate an increased autophagy flux whereas high levels of p62 occur in case of a disrupted enzymatic degradation of autophagosome cargo [30]. The similarity of the expression pattern of p62 and LC3 under obatoclax and CQ treatment further argues in favor of a late stage autophagy inhibition induced by obatoclax.

Interestingly, LC3 is not only upregulated on the protein level. On the transcriptional level we observed an upregulation of LC3 mRNA. Little is known regarding transcriptional regulation of autophagy. LC3 mRNA is upregulated upon obatoclax treatment and, to an even greater extent, in starved cells. Even not fully understood yet, transcriptional regulation has recently moved into research focus [42, 43] The transcription factor E2F has been shown to be a mediator of LC3 upregulation [44]. In nasopharyngeal carcinoma, there is some evidence for an involvement of the JNK-pathway in LC3 transcription [45]. Nevertheless, the presented experimental set up is unable to distinguish between a compensatory upregulation as a consequence of an autophagy blockade or a direct impact of obatoclax on transcription.

Subcellular localization of LC3 and the cytosolic LC3I/II ratio has been widely used to visualize and quantify autophagy [46, 47]. Nevertheless, choosing the right assay to monitor and asses autophagy has been a matter of debate [30, 48, 49]. We applied immunohistochemistry and fluorescence microscopy to analyze subcellular distribution of autophagy proteins p62 and LC3. Staining for LC3 revealed a weak expression in CRC cells in full supplemented conditions. Upon obatoclax treatment, a fundamental increase of LC3 can be observed in a homogenous cytosolic and nucleic fashion. The translocation of LC3 to the nucleus as well as its interactions with nucleic proteins has been recently described. Solely the cytosolic fraction of LC3 after lipidization mediates autophagy initiation, whereas the biological relevance of nucleic LC3 remains elusive [49]. Immunofluorescence for LC3 showed a strong expression but rather perinucleic distribution after obatoclax treatment.

P62 is a scaffold protein involved in a variety of cellular processes including NF-kB-signaling [31, 50, 51]. Recently, a correlation of p62 expression with clinicopathologic parameters has been shown for prostate cancer and gastrointestinal carcinoma [52, 53]. Under obatoclax treatment we observe an accumulation of p62, which is indicative for a late stage autophagy inhibition, since the scaffold protein is trapped in the autophagic vesicle.

This hypothesis is underpinned by our immunohistochemical findings. Indeed, control cells showed a homogenous cytosolic staining, turning into a granular stain upon obatoclax treatment. Since the specificity of p62 for autophagy is limited, an interaction of obatoclax with NF-kB signaling or oxygen signaling should be addressed in future studies [31, 54].

The decent mechanism by which obatoclax regulates autophagy remains elusive. Here, we show that obatoclax is capable of modulating autophagy even in the absence of Beclin-1. Autophagy initiation is crucially regulated by Beclin-1 [39, 55]. We observed that obatoclax treatment caused LC3 accumulation in CRC cells after Beclin-1 knockdown to the same extent as in control cells. Beclin-1’s interactions with Bcl-2 proteins have been reported to play a role in autophagy regulation [56, 57].

Obatoclax seems to interfere with autophagy independently of the Beclin-1 complex. Therefore we further investigated autophagy relevant proteins with regard to implications for obatoclax. Atg7 belongs to the ubiquitin like conjugation system (E1-like enzyme) mediating early steps of phagophore formation. Atg12 is conjugated to Atg5 and plays an important role in the conjugation of LC3 to phosphatidylethanolamine (E3-like enzyme) [58, 59]. Strikingly, RNA interference leading to a knockdown of the respective protein (Atg7 and Atg12) left LC3 activation unaltered. Taken together, our data indicate a regulative impact of obatoclax on autophagy independently of the cascaded and canonical autophagy pathway. By contrast, others reported Beclin-1 and Atg5 dependent effects of obatoclax [59]. Liang and coworkers propose an autophagy inducing effect of obatoclax, which cannot be confirmed by our data. In line with our presented findings in colorectal cancer, a Cathepsin dependent inhibition of autophagosomal lysis by obatoclax has been reported in breast cancer [60]. Interestingly, knockout of Atg7 abolished LC3 processing but failed to prevent obatoclax induced death in lung cancer cells [61]. Furthermore, obatoclax has been recently linked to the emerging concept of necroptosis [62]. In addition, endoplasmatic reticulum stress and reactive oxygen species may contribute to obatoclax effects [63, 64].

Next, we decided to investigate Mcl-1 in the context of obatoclax and autophagy, since Mcl-1 sparing ABT737 failed to inhibit autophagy. The interaction of Mcl-1 and Beclin-1 is crucial for chemotherapy resistance in leukemic B cells [65]. Mcl-1 has been described as key factor for a governed resistance towards obatoclax [66]. Interestingly, knockdown of Mcl-1 markedly suppressed LC3I level and decreased the LC3I/II ratio in cells treated with obatoclax. A regulatory role of Mcl-1 in autophagy signaling has not yet been described mechanistically and deserves further attention.

## Conclusion

In summary, our data prove that autophagy inhibition represents a potent approach to inhibit CRC cell growth in starving conditions reflecting a common phenomenon in growing tumor tissues. Furthermore, we demonstrate that pan-Bcl-2 inhibition by obatoclax utilizes autophagy inhibition as a main effector. This late stage autophagy inhibition is independent of canonical autophagy signaling pathways. Finally, our data indicate a decisive role of Mcl-1 in autophagy regulation, which needs further attention in future studies.

## Additional files

Additional file 1: Figure S1. Chloroquine induces apoptosis in starving CRC cells via autophagy inhibition. A) HT29 and SW480 cells were grown in full supplemented or reduced medium to induce starvation. Cells were treated with 20 μM Chloroquine for 48 h and apoptosis induction was subsequently assessed by flow cytometry. Values are expressed as mean ± SD. B) HT29 and SW480 cells were treated with escalating Chloroquine doses in full supplemented medium for 24 h. Representative Western blots for p62 and LC3 I/II was performed. Hypoxia (1% O_2_) and Staurosporine (2 μM, 6 h) served as control. Tubulin served as loading control. Assays were done in triplicates. C) Fluorescence microscopy in HT29 with vital Lysotracker dye after 48 h Chloroquine (20 μM) treatment versus mock treated cells. DMSO as a vesicle. *** = *p* < 0.001. (PDF 5657 kb) Additional file 2: Figure S2. Obatoclax is a very late stage autophagy inhibitor in CRC cells and allows acidification of autophagosomes. Cells were treated with either Chloroquine (30 μM, middle) or obatoclax (0.25 μM, lower) for 48 h. Acridine orange was applied. Green dots indicate unprotonated (left panel) and red dots (middle panel) protonated Acridine Orange. The right panel shows a merged overlay. Pictures are representative for three independent experiments. AO = Acridine Orange. (PDF 6121 kb)
