# Supplementary figures and images for: Pan-Bcl-2 inhibitor Obatoclax is a potent late stage autophagy inhibitor in colorectal cancer cells independent of canonical autophagy signaling
Source: BMC Cancer. 2015 Nov 19;15:919. doi: 10.1186/s12885-015-1929-y (PMC4653869; doi:10.1186/s12885-015-1929-y)

Fig. S1

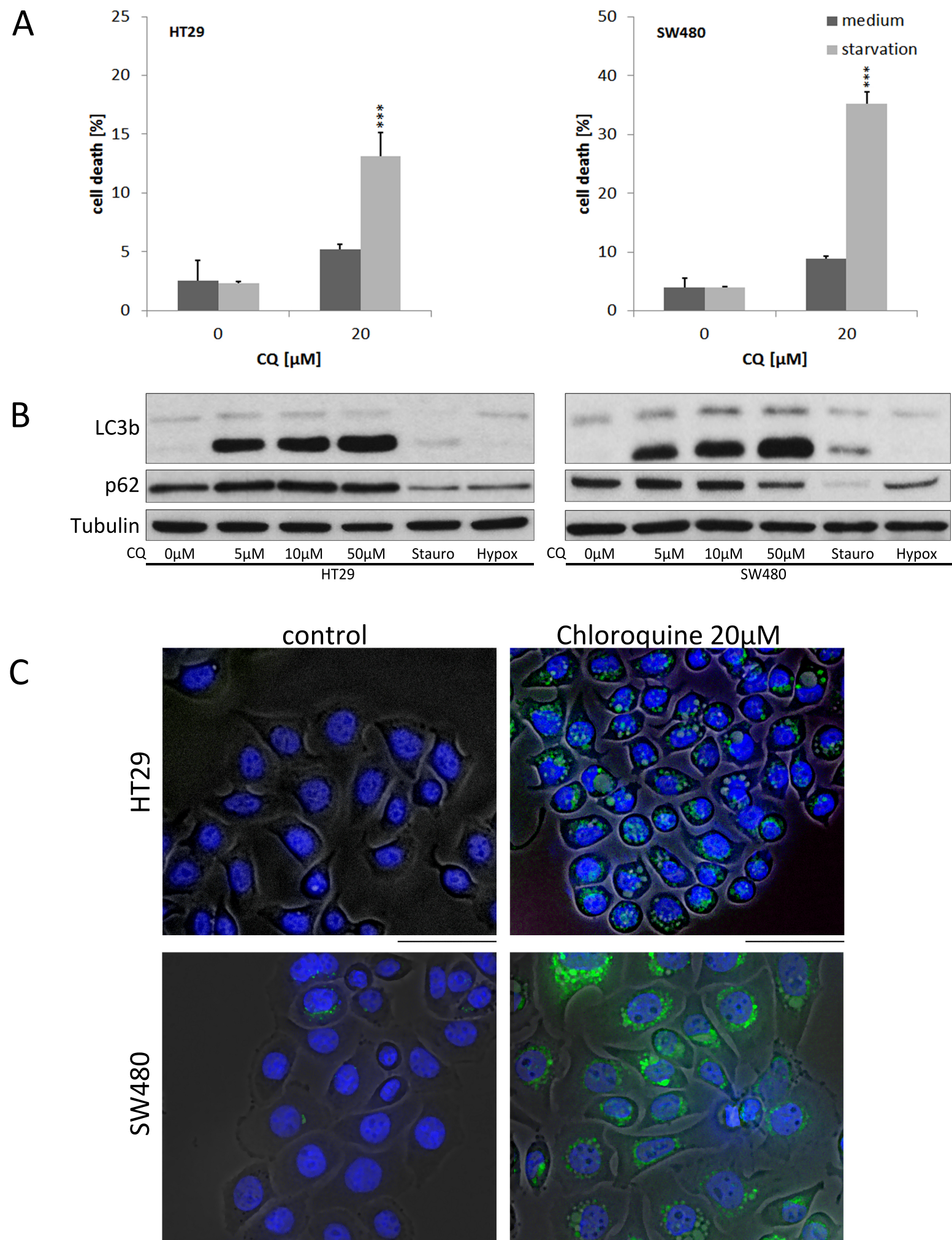

Supplement: Additional file 1: Figure S1. — Chloroquine induces apoptosis in starving CRC cells via autophagy inhibition. A) HT29 and SW480 cells were grown in full supplemented or reduced medium to induce starvation. Cells were treated with 20 μM Chloroquine for 48 h and apoptosis induction was subsequently assessed by flow cytometry. Values are expressed as mean ± SD. B) HT29 and SW480 cells were treated with escalating Chloroquine doses in full supplemented medium for 24 h. Representative Western blots for p62 and LC3 I/II was performed. Hypoxia (1% O2) and Staurosporine (2 μM, 6 h) served as control. Tubulin served as loading control. Assays were done in triplicates. C) Fluorescence microscopy in HT29 with vital Lysotracker dye after 48 h Chloroquine (20 μM) treatment versus mock treated cells. DMSO as a vesicle. *** = p < 0.001. (PDF 5657 kb) [file 12885_2015_1929_MOESM1_ESM.pdf]

Fig. S2

AO unprotonated

AO protonated

merge

control

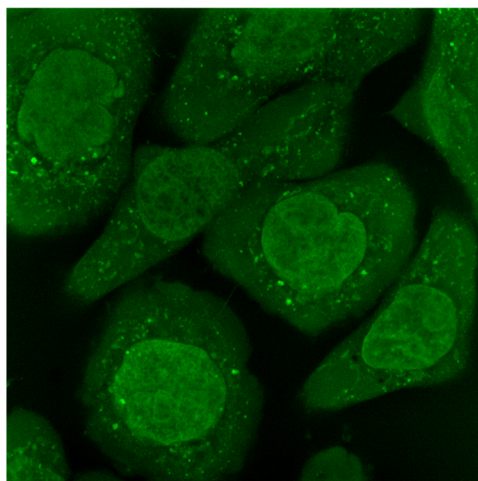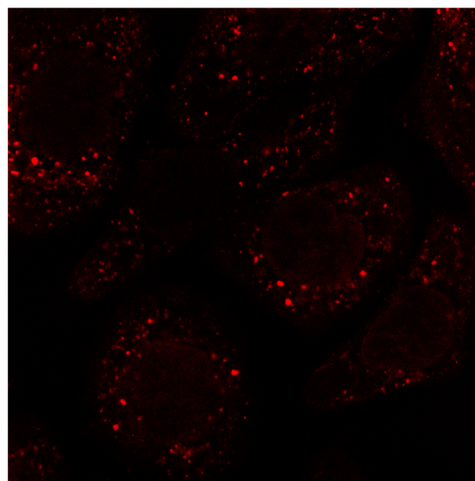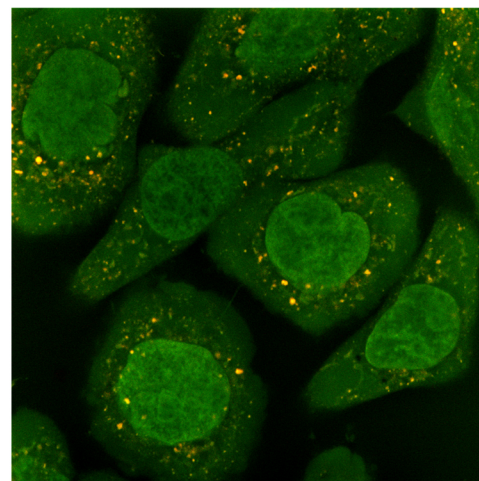

Chloroquine 30 $\mu$ M

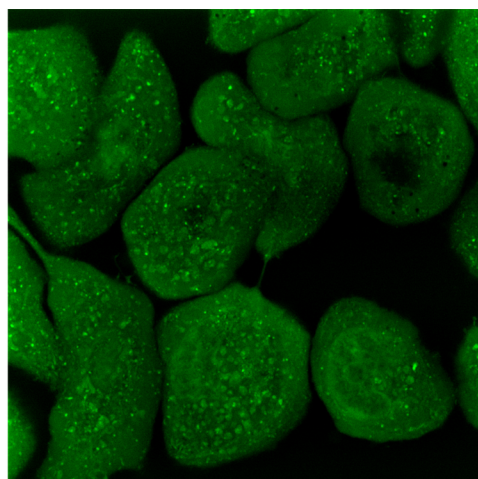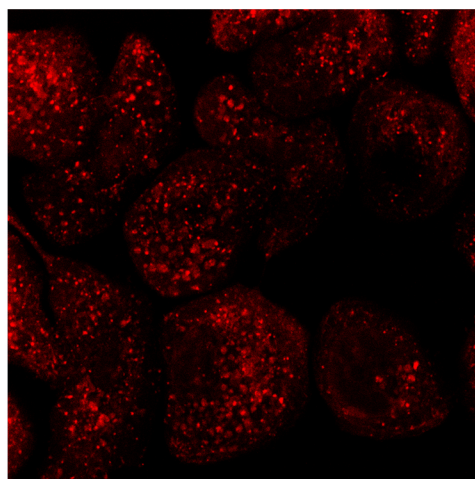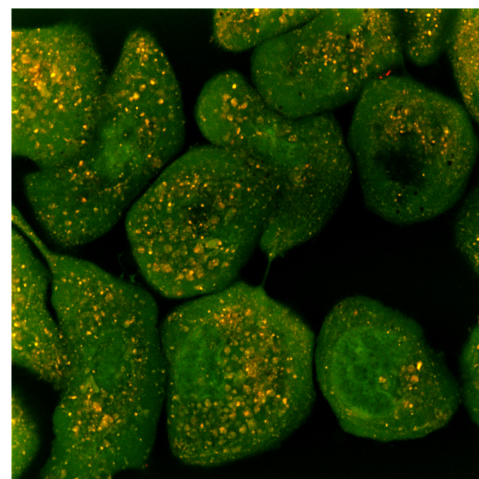

Obatoclax 0.25  $\mu$ M

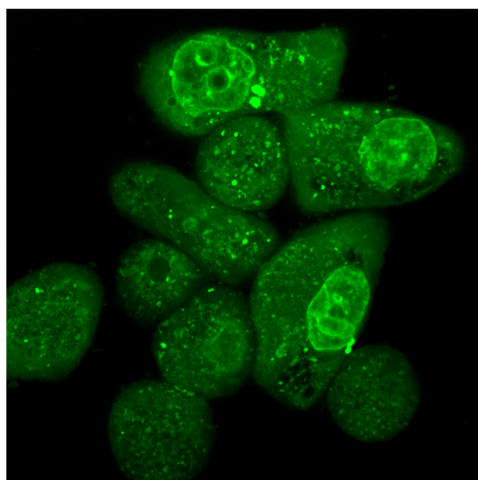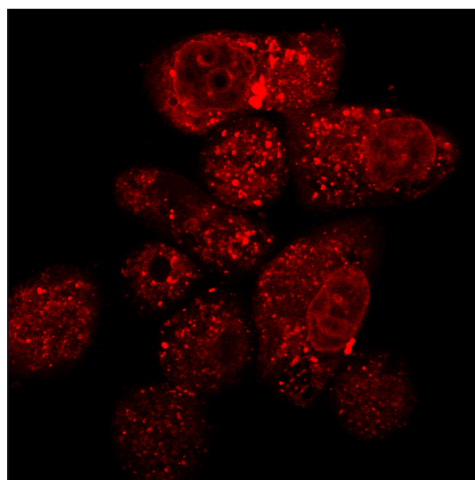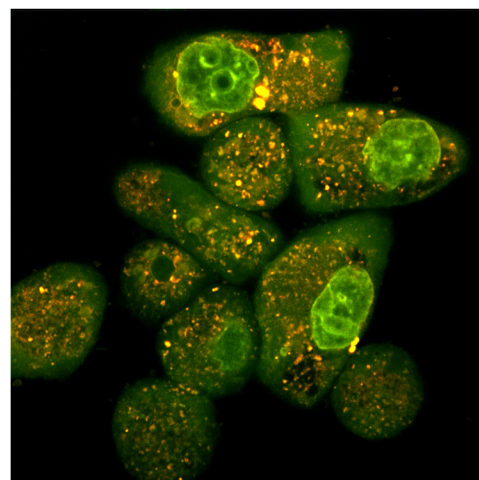

Supplement: Additional file 2: Figure S2. — Obatoclax is a very late stage autophagy inhibitor in CRC cells and allows acidification of autophagosomes. Cells were treated with either Chloroquine (30 μM, middle) or obatoclax (0.25 μM, lower) for 48 h. Acridine orange was applied. Green dots indicate unprotonated (left panel) and red dots (middle panel) protonated Acridine Orange. The right panel shows a merged overlay. Pictures are representative for three independent experiments. AO = Acridine Orange. (PDF 6121 kb) [file 12885_2015_1929_MOESM2_ESM.pdf]
